# Supplementary material for: C. elegans SSNA-1 is required for the structural integrity of centrioles and bipolar spindle assembly
Source: Nat Commun. 2025 Jun 5;16:5220. doi: 10.1038/s41467-025-59939-0 (PMC12141670; doi:10.1038/s41467-025-59939-0)

***C. elegans* SSNA-1 is required for the structural integrity of centrioles and bipolar spindle assembly**

Table S1. *C. elegans* strains used in this study

N2	Wild type
EU3000	*sas-7(or1940[gfp::sas-7]) III; ItIs37[pie-1p::mCherry::his-58 + unc-119(+)] IV*
GZ1966	*sas-1(is6[sas-1::3xFlag]) III*
OC210	*fem-1(hc17) IV*
OC779	*bsSi15[pKO109: spd-2p-spd-2::mCherry::spd-2 3'-utr, unc-119(+)] I; bsSi30[pCW9: unc-119(+) pcdk-11.2::sfgfp::his-58::cdk-11.2 3' utr] II; unc-119(ed3) III*
OC786	*zyg-1(bs212[HA::zyg-1]) II*
OC908	*bsSi30[pCW9: unc-119(+) pcdk-11.2::sfgfp::his-58::cdk-11.2 3' utr] II; bsIs20[pNP99: unc-119(+) tbb-1p::mCherry::tbb-2::tbb-2 3'-utr]; bsIs2[pCK5.5: Ppie-1::gfp::spd-2]*
OC953	*sas-6(bs175[sas-6::HA]) IV*
OC955	*sas-4(bs177[HA::sas-4]) III*
OC966	*sas-4(bs186[spot::sas-4]) III*
OC994	*sas-4(bs195[sas-4::gfp]) III*
OC1002	*zyg-1(bs197[zyg-1::spot]) II*
OC1013	*bsSi30[pCW9: unc-119(+) pcdk-11.2::sfgfp::his-58::cdk-11.2 3' utr] II; bsIs20[pNP99: unc-119(+) tbb-1p::mCherry::tbb-2::tbb-2 3'-utr]; bsIs2[pCK5.5: Ppie-1::gfp::spd-2]; ssna-1(bs182)/dpy-9(tm9713) kvs-5(tmIs1245)] IV*
OC1014	*ssna-1(bs182)/dpy9(tm9713) kvs5(tmIs1245) IV*
OC1018	*ssna-1(bs206[ssna-1::spot]) IV*
OC1020	*ssna-1(bs182)/ears-2(ve631[LoxP + myo-2p::gfp::unc-54 3' UTR + rps-27p::neoR::unc-54 3' UTR + LoxP]); fem-1(ht17ts) IV*
OC1021	*zyg-1(bs197[zyg-1::spot] II; ssna-1(bs182) IV*
OC1040	*sas-4(bs195[sas-4::gfp]) III; ssna-1(bs206[ssna-1::spot]) IV*
OC1046	*ssna-1(bs182)/dpy9(tm9713) kvs5(tmIs1245) IV*

OC1047	*zyg-1(it25) II*
OC1048	*zyg-1(it25) II; ssna-1(bs182)/dpy9(tm9713) kvs5(tmIs1245) IV*
OC1049	*ssna-1(bs218[ssna-1::C-tag]) IV*
OC1052	*ssna-1(bs220[Δ100-105])/dpy9(tm9713) kvs5(tmIs1245) IV*
OC1068	*ssna-1(bs222[G7X])/dpy9(tm9713) kvs5(tmIs1245) IV*
OC1070	*ssna-1(bs224[Δ2-22])/dpy9(tm9713) kvs5(tmIs1245) IV*
OC1072	*ssna-1(bs231[Δ2-17])/dpy9(tm9713) kvs5(tmIs1245) IV*
OC1076	*ssna-1(bs235[Δ2-18])/dpy9(tm9713) kvs5(tmIs1245) IV*
OC1078	*ssna-1(bs237[Δ2-18::spot])/dpy9(tm9713) kvs5(tmIs1245) IV*
OC1126	*spd-5(vie26[gfp::spd-5 +loxP]) I; ssna-1(bs206[ssna-1::spot]) IV*
OC1129	*spd-5(vie26[gfp::spd-5 +loxP]) I; ltIs37[pAA64: unc-119(+) pie-1-mcherry-his58]*
OC1130	*spd-5(vie26[gfp::spd-5 +loxP]) I; ssna-1(bs182) IV; ltIs37[pAA64: unc-119(+) pie-1-mcherry-his58]*
OC1132	*ssna-1(bs243[ssna-1::wrmScarlet) IV*
OC1134	*sas-6(bs190[sas-6::spot]) IV*
OC1135	*ssna-1(bs182), sas-6(bs190[sas-6::spot]) IV*
OC1138	*ssna-1(bs182)/ears-2(ve631[LoxP + myo-2p::gfp::unc-54 3' UTR + rps-27p::neoR::unc-54 3' UTR + LoxP]) IV*
OC1139	*ssna-1(bs243[ssna-1::wrmScarlet]) IV; ltSi560[pPLG014; Pmex-5::gfp::his-11::tbb2_3'UTR, tbg-1::gfp::tbb-2_3'UTR; cb-unc-119(+)] V*
OC1166	*sas-1(t1476, bs272) III*
OC1177	*bsSi15[pKO109: spd-2p-spd-2::mCherry::spd-2 3'-utr, unc-119(+)] I; bsSi30[pCW9: unc-119(+) pcdk-11.2::sfgfp::his-58::cdk-11.2 3' utr], zyg-1(it25) II*
OC1178	*bsSi15[pKO109: spd-2p-spd-2::mCherry::spd-2 3'-utr, unc-119(+)] I; bsSi30[pCW9: unc-119(+) pcdk-11.2::sfgfp::his-58::cdk-11.2 3' utr], zyg-1(it25) II; ssna-1(bs182)/ears-2(ve631[LoxP + myo-2p::GFP::unc-54 3' UTR + rps-27p::neoR::unc-54 3' UTR + LoxP]) IV*
OC1183	*bsSi15[pKO109: spd-2p-spd-2::mCherry::spd-2 3'-utr, unc-119(+)] I; bsSi30[pCW9: unc-119(+) pcdk-11.2::sfgfp::his-58::cdk-11.2 3' utr] II; ssna-1 (bs182)/ears-2(ve631[LoxP + myo-2p::gfp::unc-54 3' UTR + rps-27p::neoR::unc-54 3' UTR + LoxP]) IV*

OC1205	*sas-5(bs199[sas-5::spot]) V*
OC1207	*ssna-1(bs182)/ears-2(ve631[LoxP + myo-2p::gfp::unc-54 3' UTR + rps-27p::neoR::unc-54 3' UTR + LoxP]) IV; sas-5(bs199[sas-5::spot]) V*
OC1209	*sas-7(or1940[gfp::sas-7]) III; ssna-1(bs182)/ears-2(ve631[LoxP + myo-2p::gfp::unc-54 3' UTR + rps-27p::neoR::unc-54 3' UTR + LoxP]), ItIs37[pie-1p::mCherry::his-58 + unc-119(+)] IV*
OC1230	*bsIs55[pKO109: spd-2p-spot::spd-2 3'-utr, unc-119(+)] I; bsSi30[pCW9: unc-119(+) pcdk-11.2::sfgfp::his-58::cdk-11.2 3' utr] II; unc-119(ed3) III*
OC1264	*sas-4(bs186[spot::sas-4]) III; ssna-1(bs182)/ears-2(ve631[LoxP + myo-2p::gfp::unc-54 3' UTR + rps-27p::neoR::unc-54 3' UTR + LoxP]) IV*
OC1265	*sas-4(bs195[sas-4::gfp]) III; ssna-1(bs182)/ears-2(ve631[LoxP + myo-2p::gfp::unc-54 3' UTR + rps-27p::neoR::unc-54 3' UTR + LoxP]) IV*
OC1335	*sas-1(t1476, bs272) III; ssna-1(bs182)/ears-2(ve631[LoxP + myo-2p::gfp::unc-54 3' UTR + rps-27p::neoR::unc-54 3' UTR + LoxP]) IV*
OC1344	*ssna-1(bs356[R18E::spot]) IV*
OC1410	*sas-1(bs396[HA::sas-1]) III; ssna-1(bs182)/ears-2(ve631[LoxP + myo-2p::GFP::unc-54 3' UTR + rps-27p::neoR::unc-54 3' UTR + LoxP]) IV*
OC1412	*sas-1(bs398[spot::sas-1]), sas-7(or1940[gfp::sas-7]) III; ssna-1(bs182)/ears-2(ve631[LoxP + myo-2p::GFP::unc-54 3' UTR + rps-27p::neoR::unc-54 3' UTR + LoxP]) IV*
OC1414	*sas-1(bs398[spot::sas-1]), sas-7(or1940[gfp::sas-7]) III*
OC1419	*bsIs60[spd-2p::ssna-1::spot::spd-2 3'-utr] I*
OC1420	*bsIs60 [spd-2p::ssna-1::spot::spd-2 3'-utr] I; ssna-1(bs182)/ears-2(ve631[LoxP + myo-2p::GFP::unc-54 3' UTR + rps-27p::neoR::unc-54 3' UTR + LoxP]) IV*
OC1421	*bsIs60[spd-2p::ssna-1::spot::spd-2 3'-utr] I; sas-1(t1476, bs272) III*

Table S2: crRNAs and repair templates used in this study

<table>
<tr><th>Allele</th><th>Background</th><th>crRNA (5’ -> 3’)</th><th>Repair template (5’ -> 3’)</th></tr>
<tr><td>bs199</td><td>N2</td><td>5’-TTCGTGAAAAATACGCTCGC-3’</td><td>CTGAACGAGAACGCCGTATTCGTGAAA
AATACGCTCGCAGAAAACCAGACCGTG
TCCGTGCCGTCTCCCACTGGTCCTCCTG
ATATCAAATGTGTTTAACTCTTGACGTT
TTAAA</td></tr>
<tr><td>bs206</td><td>N2</td><td rowspan="8">5’-CTTTGTGCGCAAAGAGTATC-3’

5’-GATATATTTACAGGCATTTT-3’</td><td rowspan="5">GCAAAAGACGTTGGTGGACTTTGTGCG
CAAAGAGTATCAAGATACGAAACATCA
GAAATATCCAGACCGTGTCCGTGCCGT
CTCCCACTGGTCCTCCTGAACTCTGAAC
AACTGTCTCCCAAAAATGCCTGTAAAT
ATATCAATTATCGACATAACTTC</td></tr>
<tr><td>bs233</td><td>bs231</td></tr>
<tr><td>bs237</td><td>bs235</td></tr>
<tr><td>bs249</td><td>bs217</td></tr>
<tr><td>bs357</td><td>bs286</td></tr>
<tr><td>bs218</td><td>N2</td><td>GCAAAAGACGTTGGTGGACTTTGTGCG
CAAAGAGTATCAAGATACGAAACATCA
GAAATATGAACCGGAAGCGTGAACTCT
GAACAACTGTCTCCCAAAAATGCCTGT
AAATATATCAATTATCGACATAACTTC</td></tr>
<tr><td rowspan="2">bs242</td><td rowspan="2">N2</td><td>GCAAAAGACGTTGGTGGACTTTGTGCG
CAAAGAGTATCAAGATACGAAACATCA
GAAATATGGATCCGCCGGATCCGCCGC
CGGATCCGGAGAGTTCGTCAGCAAGGG
AGAGGCAGTTATCAAGGAGTTCATGCG
TTTCAAGGTCCACATGGA</td></tr>
<tr><td>TATCAAGGAGTTCATGCGTTTCAAGGTC
CACATGGAGGGATCCATGACCGAGGGA
CGTCACTCCACCGGAGGAATGGACGAG
CTCTACAAGTGAACTCTGAACAACTGT
CTCCCAAAAATGCCTGTAAATATATCA
ATTATCGACATAACTTC</td></tr>
<tr><td>bs243</td><td>bs242</td><td>5’-CATGGAGGGATCCATGACCG-3’</td><td>GTCAGCAAGGGAGAGGCAGTTATCAAG
GAGTTCATGCGTTTCAAGGTCCACATG
GAGGGATCCATGAACGGACACGAGTTC
GAGATCGAGGGAGAGGGAGAGGGACG
TCCATACGAGGGAACCCAAACCGCCAA
GCTCAAGGTCACCAAGGGAGGACCACT
CCCATTCTCCTGGGACATCCTCTCCCCA
CAATTCATGTACGGATCCCGTGCCTTCA
CCAAGCACCCAGCCGACATCCCAGACT
ACTACAAGCAATCCTTCCCAGAGGGAT
TCAAGTGGGAGCGTGTCATGAACTTCG
AGGACGGAGGAGCCGTCACCGTCACCC
AAGACACCTCCCTCGAGGACGGAACCC</td></tr>
</table>

			TCATCTACAAGGTCAAGCTCCGTGGAA<br>CCAACTTCCCACCAGACGGACCAGTCA<br>TGCAAAAGAAGACCATGGGATGGGAG<br>GCCTCCACCGAGCGTCTCTACCCAGAG<br>GACGGAGTCCTCAAGGGAGACATCAAG<br>ATGGCCCTCCGTCTCAAGGACGGAGGA<br>CGTTACCTCGCCGACTTCAAGACCACCT<br>ACAAGGCCAAGAAGCCAGTCCAAATGC<br>CAGGAGCCTACAACGTCGACCGTAAGC<br>TCGACATCACCTCCCACAACGAGGACT<br>ACACCGTCGTCGAGCAATACGAGCGTT<br>CCGAGGGACGTCACTCCACCGGAGGAA<br>TGGACGAGCTCTACAAG
bs182	N2	5’-TAGAATCATGCATTTGCATT-3’<br><br>5’-CTTTGTGCGCAAAGAGTATC-3’	TTCGTATTTGAACAATTACTGACTAATT<br>TCCTCCGAATGCAAATGCATGATTCTAG<br>AACAAAAAAAACATCAGAAATATTGAA<br>CTCTGAACAACTGTCTC
bs215	N2	5’-CTGTGAGACGGCGTTCCTCT-3’	GAAGATAAACATTTATAGTAATATTTC<br>AGACATCAAGCGCTCAGAGAGGAACGC<br>CGTCTCACAGAATCGTCGATTCGAAAA<br>ATG
bs216	bs215	5’-AGACATCCAAGCGCTCGCGG-3’	GTAAGAGCAAATTGAAGATAAACATTT<br>ATAGTAATATTTCAGACATCCAAGCGC<br>TCGCGGAAGAACGCCGTCTCACAGAAT<br>CGTCGATTCGAAAAATGGA
bs217			GTAAGAGCAAATTGAAGATAAACATTT<br>ATAGTAATATTTCAGACATCCAAGCGC<br>TCGCGGCAGCACGCCGTCTCACAGAAT<br>CGTCGATTCGAAAAATGGAAA
bs220	N2	5’-CTTTGTGCGCAAAGAGTATC-3’	GCAAAAGACGTTGGTGGACTTTGTGCG<br>CAAAGAGTATCAAGATTGAACTCTGAA<br>CAACTGTCTCCCAAAAATGCCTGT
bs222	N2	5’-AAAATGTCTTCTCGATCTAC-3’	TGCATGATTCTAGAACAAAAAATGTCT<br>TCTCGATCTACATGAAGCTTTGATGAAA<br>TATCACAGTGTAAGAGCAAATT
bs224	bs215	5’-AAATGTCTTCTCGATCTAC-3’<br><br>5’-AGACATCCAACGTCTCCGAG-3’	CCGAATGCAAATGCATGATTCTAGAAC<br>AAAAAATGCGCCGTCTCACAGAATCGT<br>CGATTCGAAAAATGGA

bs231	N2	5’-AAAATGTCTTCTCGATCTAC-3’<br>5’-CTGTGAGACGGCGTTCCTCT-3’	CCGAATGCAAATGCATGATTCTAGAAC AAAAAATGCGTCTCAGAGAGGAACGCC GTCTCACAGAATCGTCGATTCGAAAAA
bs235	N2		CCGAATGCAAATGCATGATTCTAGAAC AAAAAATGCTCAGAGAGGAACGCCGTC TCACAGAATCGTCGATTCGAAAAA
bs284	N2		TGCATGATTCTAGAACAAAAAATGTCT TCTCGAAGCACAGGAAGCTTTGATGAA ATATCACAGGGTAAGAGCAAATTGAAG ATAAACATTTATAGTAATATTTCAGAG ATCCAACGTCTCAGAGAGGAACGCCGT CTCACAGAATCGTCGATTCGAAAAA
bs286	N2	5’-CTGTGAGACGGCGTTCCTCT-3’	GAAGATAAACATTTATAGTAATATTTC AGACATCCAAGAGCTCAGAGAGGAACG CCGTCTCACAGAATCGTCGATTCGAAA AATG
bs312	bs284	5’-CTTTGTGCGCAAAGAGTATC-3’	CGCAAAAGACGTTGGTGGACTTTGTGC GCAAAGAGGAGCAGGATACGAAACAT CAGAAATATTGAACTCTGAACAAC
bs314	bs286		
bs335	N2		
bs356	bs206	5’-AGAGGAACGCCGTCTCACAG-3’	GAAGATAAACATTTATAGTAATATTTC AGACATCCAAGAGCTCAGAGAGGAACG CCGTCTCACAGAATCGTCGATTCGAAA AATG
bs396	N2	5’-ACAATTACTGGTGCCCTACG-3’	TTTTCAAAATTTTAAAACTTCTTTCAGA ACTAATGTACCCATACGATGTTCCAGAT TACGCTAAGCCGCGTAGGGCACCAGTA ATTGTCAAAGCTTTTGATGAAAA
bs398	N2	5’-ACAATTACTGGTGCCCTACG-3’	TTTTCAAAATTTTAAAACTTCTTTCAGA ACTAATGCCAGACCGTGTCCGTGCCGT CTCCCACTGGTCCTCCAAGCCGCGTAG GGCACCAGTAATTGTCAAAGCTTTTGAT GAAAA

bsIs60	bsIs55	5’-AGTGGGAGACGGCACGGACA-3’	CATTGGCGCGCCCGTCAAATGATTAGTGTTTACGCTTTTAAAATCATTCATTTTAAAACTTTTTCTATTCTCAGCGTATTAAAAATGTCCTCCCGTTCCACCGGATCCTTCGACGAGATCTCCCAATACATCCAACGTCTCCGTGAGGAGCGTCGTCTCACCGAGTCCTCCATCCGTAAGGTAAGTTTAAACATATATATACTAACTAACCCTGATTATTTAAATTTTCAGATGGAGAAGGAGAAGTCCGACCTCAACGAGAAGGTAAGTTTAAACAGTTCGGTACTAACTAACCATACATATTTAAATTTTCAGATCGACGAGCTCACCACCCGTAAGTGCTCCGTCGACGCCCGTCTCCAAGCCGAGAACGAGCGTGCCGAGCGTCAAGACCGTGGACTCAAGGTAAGTTTAAACATGATTTTACTAACTAACTAATCTGATTTAAATTTTCAGGAGGCCGAGACCACCTACGCCAAGCTCGTCGAGTCCCAAAAGACCCTCGTCGACTTCGTCCGTAAGGAGTACCAAGACACCAAGCACCAAAAGTACCCAGATCGTGTCCGTGCCGTCTCCCACTGGTCCTCC

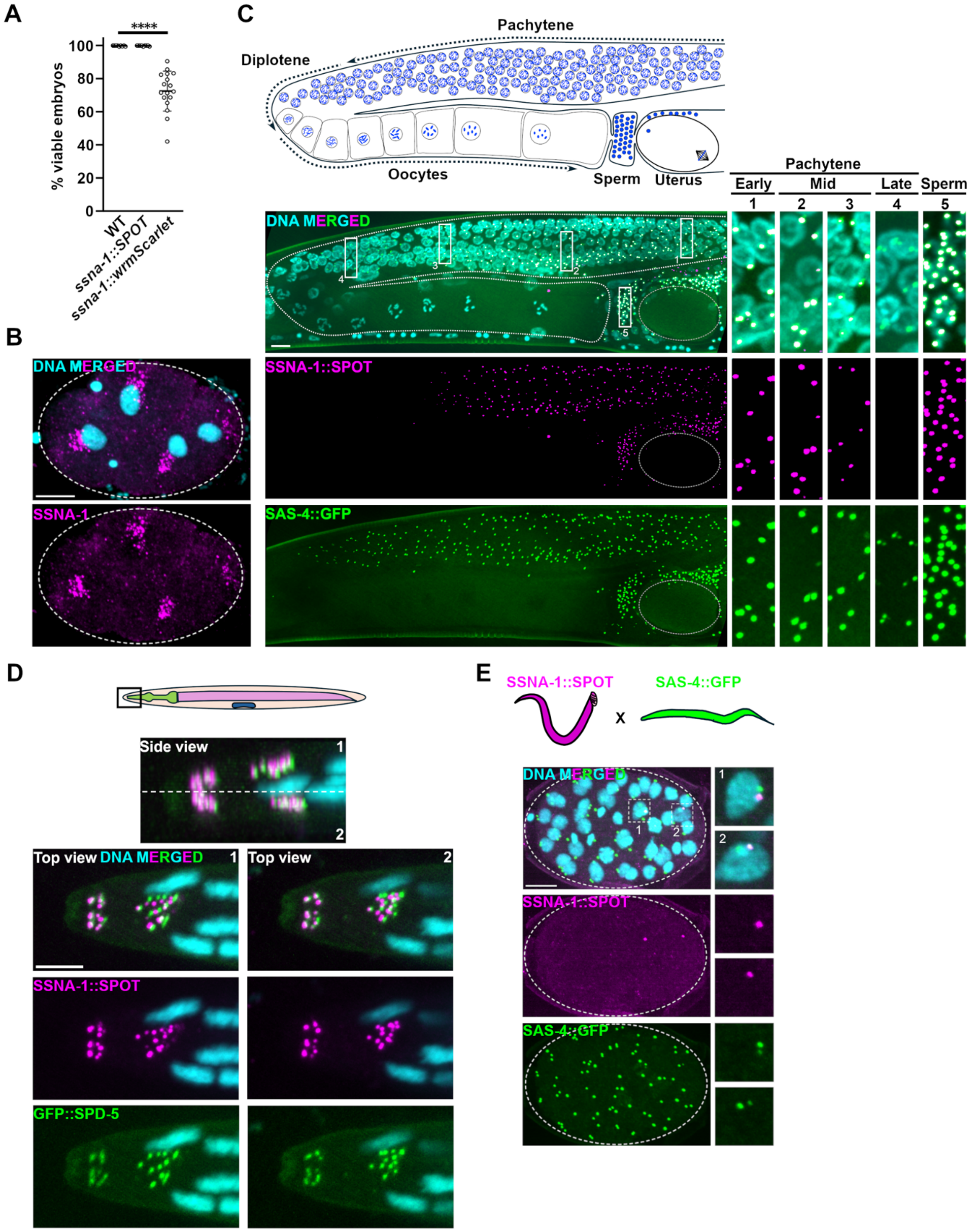
A
****
% viable embryos
100
80
60
40
20
0
WT
ssna-1::SPOT
ssna-1::wrmScarlet
C
Pachytene
Diplotene
Oocytes
Sperm
Uterus
Pachytene
Early
Mid
Late
Sperm
1
2
3
4
5
DNA MERGED
SSNA-1::SPOT
SAS-4::GFP
B
DNA MERGED
SSNA-1
D
Side view
Top view DNA MERGED
Top view
SSNA-1::SPOT
GFP::SPD-5
E
SSNA-1::SPOT
SAS-4::GFP
x
DNA MERGED
SSNA-1::SPOT
SAS-4::GFP

Fig. S1: SSNA-1 is a stable component of centrioles with broad distribution in the worm. A Quantification of embryonic viability of strains expressing different C-terminal tagged versions of endogenous SSNA-1. Each datapoint represents progeny of a single hermaphrodite. Mean and SD are shown. n= 9 (WT), 9 (*ssna-1::SPOT*), 17 (*ssna-1::wrmScarlet*). ****p<0.0001 as determined with a one-way ANOVA with Tukey's multiple comparisons test. **B** Representative image of an embryo stained for endogenous SSNA-1 and DNA. Bar = 10 μm. Experiment was performed 4 times. All embryos looked similar. **C** Top, diagram of the *C. elegans* hermaphrodite germ line showing the position of early meiotic nuclei distally and oocytes and sperm proximally. Bottom, localization of SSNA-1::SPOT in the germ line with SAS-4::GFP marking centrioles, showing that SSNA-1 is present on centrioles in immature female germ cells as well as on sperm. SSNA-1 is lost from germ cell centrioles beginning during mid-pachytene (box 3), prior to SAS-4 (box 4). Bar = 10 μm. Experiment was performed 2 times. All 14 gonads scored looked similar. **D** SSNA-1::SPOT is found adjacent to the acentriolar centrosome (marked with GFP::SPD-5) in amphid neurons of L1 larvae. The diagram illustrates the area of the worm being imaged. Bar = 10 μm. Seven worms were scored for SSNA-1::SPOT only and looked similar. Two were scored for both SSNA-1::SPOT and GFP::SPD-5 and looked similar. **E** An embryo produced by mating SSNA-1::SPOT males to SAS-4::GFP hermaphrodites stained for SAS-4::GFP (green), SSNA-1::SPOT (magenta) and DNA (cyan). Even after many cell cycles SSNA-1::SPOT remains associated with the two original sperm centrioles revealing that SSNA-1 is a stable component of centrioles. Scale bar = 10 μm. All 40 embryos scored gave identical results. Source data are provided as a Source Data file.

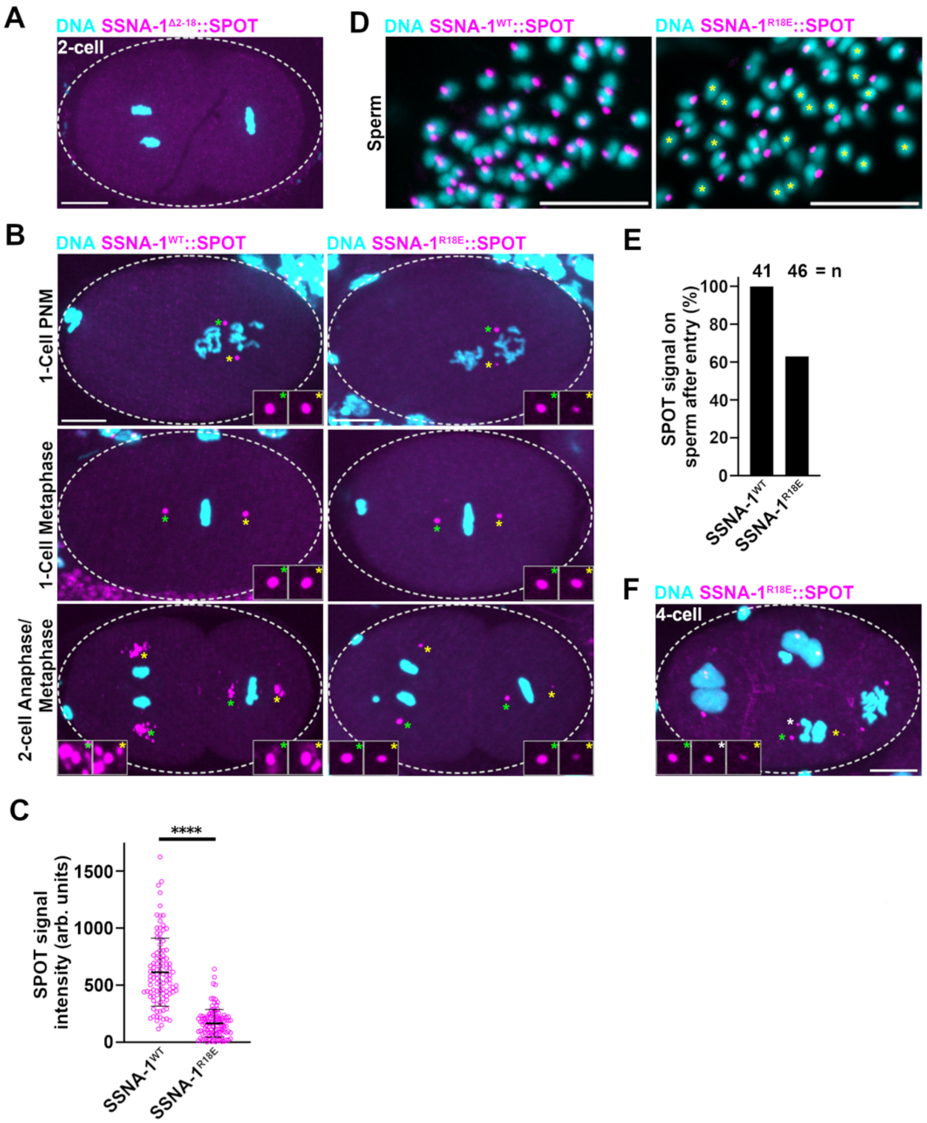

A
DNA SSNA-1Δ2-18::SPOT
2-cell
B
DNA SSNA-1WT::SPOT
DNA SSNA-1R18E::SPOT
1-Cell PNM
1-Cell Metaphase
2-cell Anaphase/ Metaphase
C
SPOT signal intensity (arb. units)
****
1500
1000
500
0
SSNA-1WT
SSNA-1R18E
D
DNA SSNA-1WT::SPOT
DNA SSNA-1R18E::SPOT
Sperm
E
41 46 = n
SPOT signal on sperm after entry (%)
100
80
60
40
20
0
SSNA-1WT
SSNA-1R18E
F
DNA SSNA-1R18E::SPOT
4-cell

Fig. S2: SSNA-1 oligomerization and MT binding are required for centriolar and satellite localization. **A** Representative image of a two-cell stage embryo expressing SSNA-1$^{\Delta 2\text{-}18}$::SPOT showing that SSNA-1 fails to localize to the centriole and satellite-like structures. Scale bar = 10 µm. All six embryos scored looked identical. **B** Localization of SSNA-1^{WT}::SPOT (left column) and SSNA-1^{R18E}::SPOT (right column) from pronuclear migration (PNM) to the late 2-cell stage in strains grown at 20°C. Full-size images are maximum intensity projections while insets are 2X magnifications of the center z plane of each centrosome indicated by colored asterisks. Scale bar = 10 µm. **C** Quantification of SSNA-1 signal intensity from B showing that the centriole levels of SSNA-1^{R18E}::SPOT are strongly reduced relative to wild-type. Each datapoint represents the intensity of a single centrosome. Mean and SD are shown. n= 112(WT), 102(SSNA-1^{R18E}). ****$p<0.0001$ as determined by an unpaired two-tailed Student's *t* test. **D** Maximum intensity projections of sperm from SSNA-1^{WT}::SPOT and SSNA-1^{R18E}::SPOT strains. Note that in the wild type, each sperm nucleus is associated with a SSNA-1::SPOT focus while many sperm in the SSNA-1^{R18E}::SPOT strain lack a focus (asterisks). Scale bar = 10 µm. Experiment was performed 4 times with similar results. **E** Quantitation showing the percentage of wild-type and R18E meiotic stage embryos that possess sperm derived centrioles that stain for SSNA-1. **F** A four-cell stage embryo expressing SSNA-1^{R18E}::SPOT grown at 25°C. Note the presence of a multipolar spindle in the EMS cell at bottom. Full-scale images are maximum intensity projections while insets are 2X magnifications of the center z plane of each centrosome as marked by colored asterisks. Scale bar = 10µm. Experiment was performed twice with similar results. Source data are provided as a Source Data file

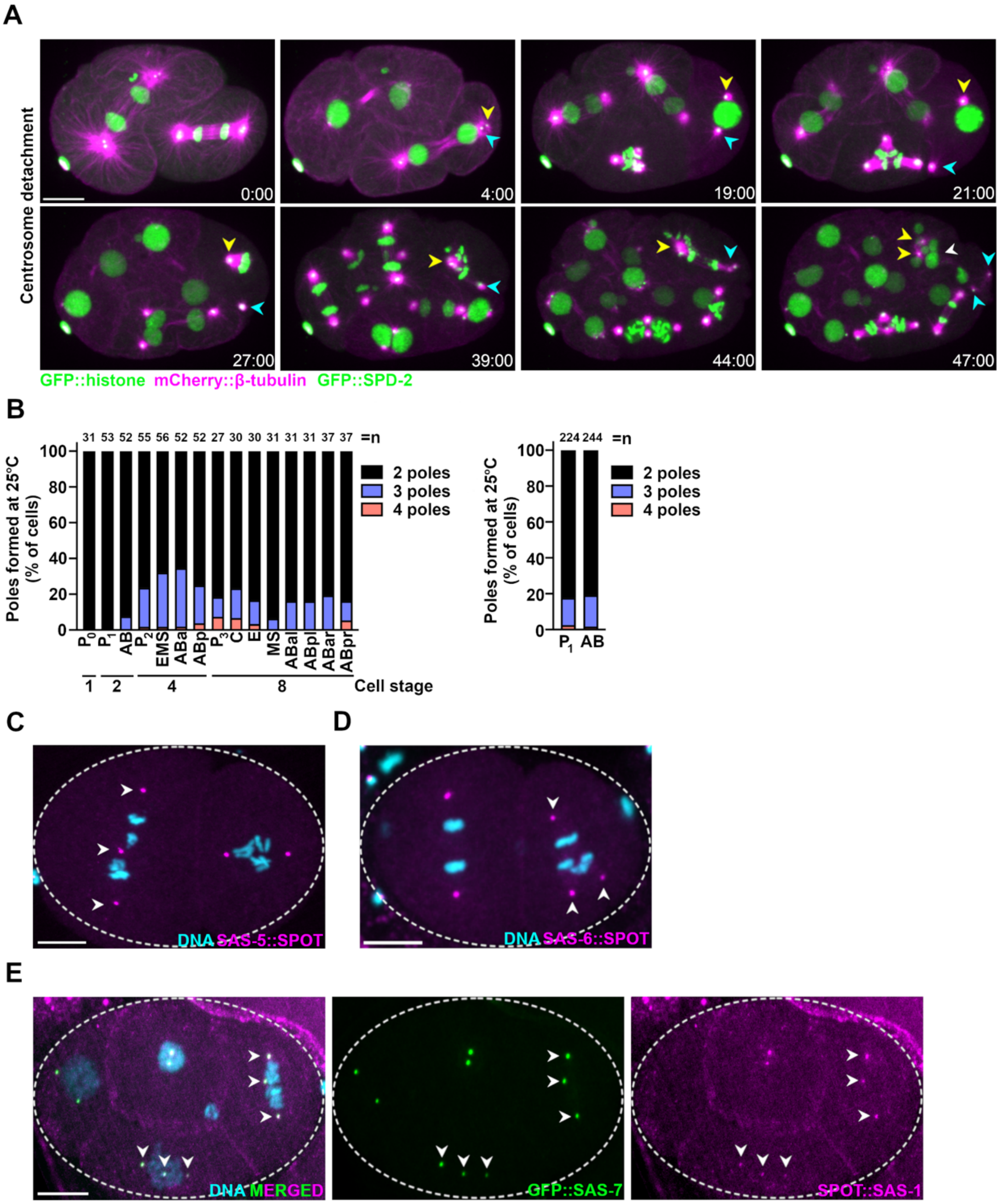
A
Centrosome detachment
0:00
4:00
19:00
21:00
27:00
39:00
44:00
47:00
GFP::histone mCherry::β-tubulin GFP::SPD-2
B
Poles formed at 25°C (% of cells)
=n
31 53 52 55 56 52 52 27 30 30 31 31 31 37 37
2 poles
3 poles
4 poles
P0 P1 AB P2 EMS ABa ABp P3 C E MS ABal ABpl ABar ABpr
Cell stage
1 2 4 8
224 244
P1 AB
C
DNA SAS-5::SPOT
D
DNA SAS-6::SPOT
E
DNA MERGED
GFP::SAS-7
SPOT::SAS-1

Fig. S3: Cell division defects during development of *ssna-1(Δ)* embryos. A Frames from a time-lapse recording of an *ssna-1(Δ)* embryo expressing GFP::SPD-2 (green); GFP::histone (green); and mCherry::β-tubulin (magenta). Yellow and cyan arrowheads indicate the two centrosomes that arise from one pole of the P_1 spindle (t=4:00). One centrosome stays associated with the P_2 nucleus (yellow) while the other (cyan) migrates to the cell periphery (t=21:00). The itinerant centrosome makes a late contribution to bipolar spindle assembly (t=44:00), but chromosome segregation defects lead to micronuclei formation (white arrowhead) (t=47:00). Note that both centrosomes duplicated. Scale bar = 10 µm. A similar detatched centrosome phenotype was observed in 9 of 57 embryos scored. **B** Left, distribution of multipolar spindle formation among the early embryonic blastomeres (n= number of cells scored). Right, percentage of cells within the P_1 and AB lineages that form multipolar spindles. **C** All poles of a multipolar spindle stain for SAS-5::SPOT (arrowheads). Scale bar = 10 µm. Experiment was performed five times with similar results. **D** All poles of a multipolar spindle stain for SAS-6::SPOT (arrowheads). Scale bar = 10 µm. Experiment was performed three times with similar results. **E** All poles of two multipolar spindles stain for both GFP::SAS-7 and SPOT::SAS-1 (arrowheads). Scale bar = 10 µm. Experiment was performed three times with similar results. Source data are provided as a Source Data file

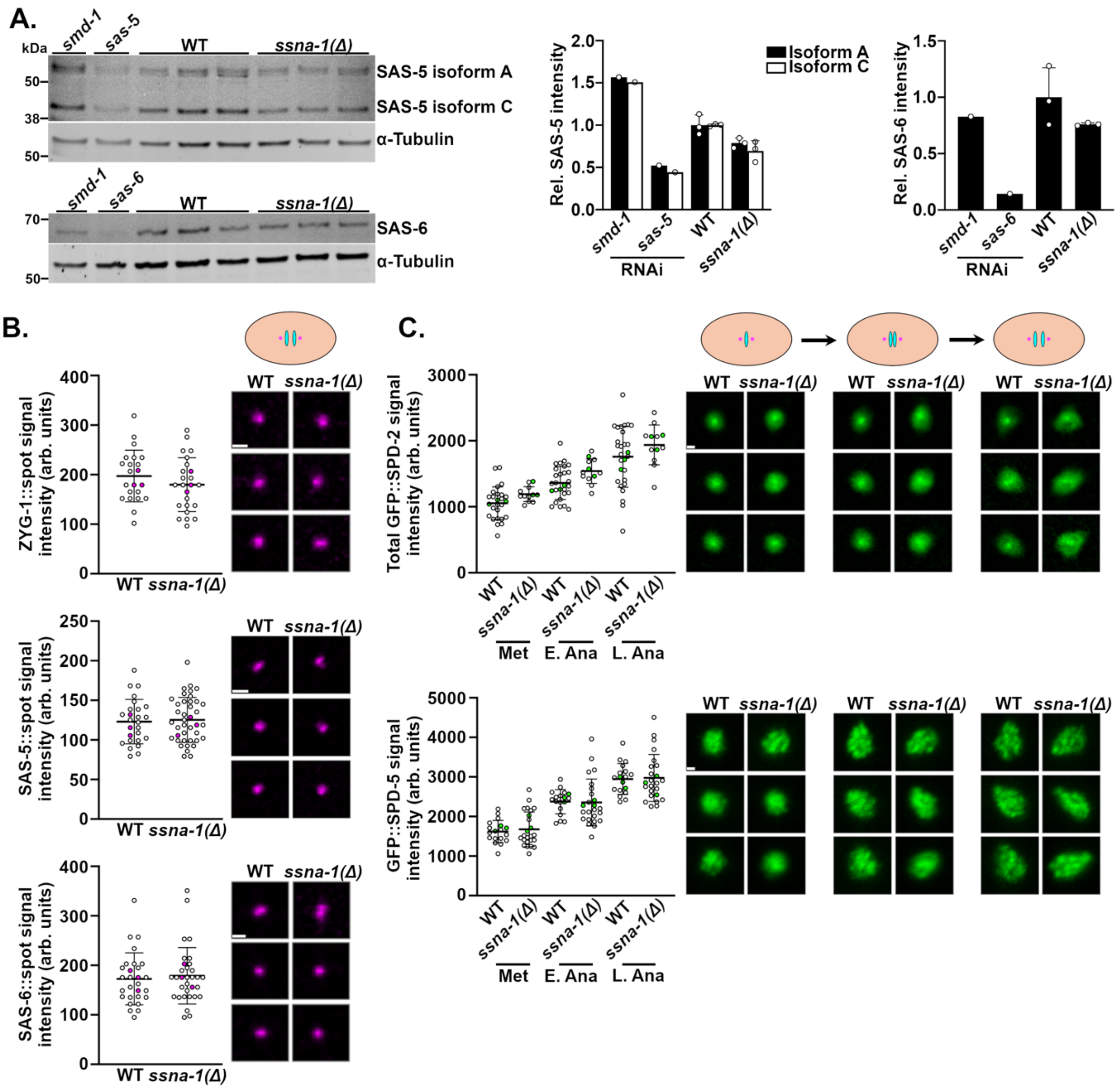
A.
kDa
smd-1
sas-5
WT
ssna-1(Δ)
SAS-5 isoform A
SAS-5 isoform C
α-Tubulin
sas-6
SAS-6
Isoform A
Isoform C
Rel. SAS-5 intensity
Rel. SAS-6 intensity
RNAi
B.
ZYG-1::spot signal intensity (arb. units)
SAS-5::spot signal intensity (arb. units)
SAS-6::spot signal intensity (arb. units)
C.
Total GFP::SPD-2 signal intensity (arb. units)
GFP::SPD-5 signal intensity (arb. units)
Met
E. Ana
L. Ana

Fig. S4: Loss of SSNA-1 is not associated with overexpression of centriole duplication factors. A Immunoblots of SAS-5 and SAS-6 levels in extracts prepared from gravid hermaphrodites (left). Quantitation of blots (right) shows the relative levels after normalization to α-tubulin. The level of each protein was measured from three independent lysates. **B** Centriole-associated levels of ZYG-1::SPOT, SAS-5::SPOT, and SAS-6::SPOT at anaphase in the zygote, as determined by quantitative immunofluorescence. Each datapoint represents an individual centrosome. Mean and SD are shown. ZYG-1: n= 22 (WT), 24 (*ssna-1(Δ)*); SAS-5: n= 26 (WT), 40 (*ssna-1(Δ)*); SAS-6: n= 28 (WT), 32 (*ssna-1(Δ)*). Scale bars = 1 μm. **C** Centrosome-associated levels of GFP::SPD-2 (top) and GFP::SPD-5 (bottom) from metaphase through anaphase in the zygote. Each datapoint represents an individual centrosome from live imaging experiments. Mean and SD are shown. SPD-2: n= WT: 26 (Meta), 28 (E. Ana), 28 (L. Ana), *ssna-1(Δ)*: 12 (Meta), 12 (E. Ana), 12 (L. Ana); SPD-5: n= WT: 18 (Meta), 18 (E. Ana), 18 (L. Ana), *ssna-1(Δ)*: 24 (Meta), 24 (E. Ana), 24 (L. Ana). In panels B and C, all differences between wild-type and *ssna-1(Δ)* mutants were found not to be significant as determined by an unpaired two-tailed Student's *t* test (Panel B) or a one-way ANOVA with Tukey's multiple comparisons test (Panel C). Images are three distinct pairs of representative centrosomes, and their intensity values are indicated as colored datapoints in each graph. Scale bars = 1 μm. Source data are provided as a Source Data file

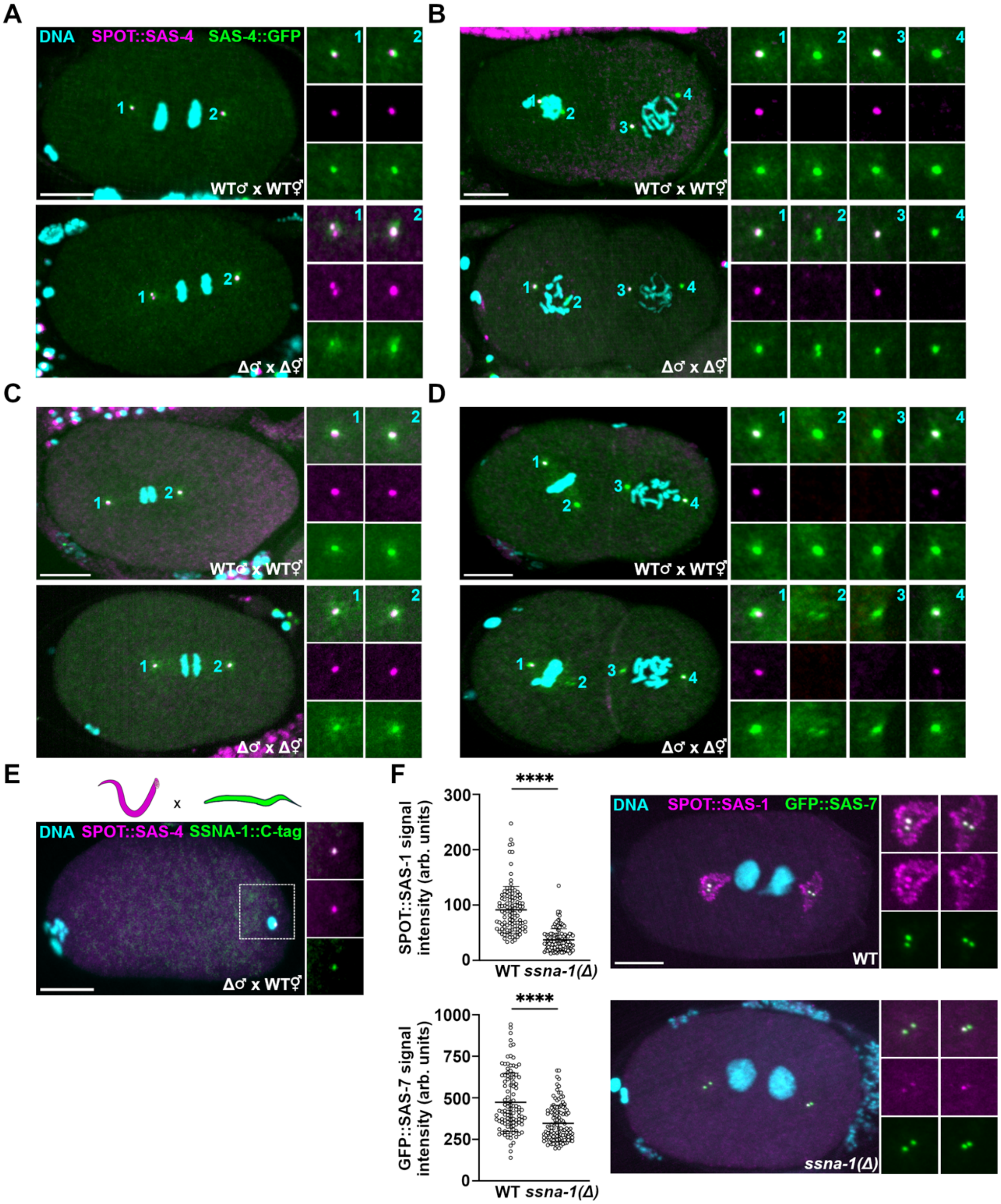
A
DNA SPOT::SAS-4 SAS-4::GFP
WT♂ x WT⚥
Δ♂ x Δ⚥
B
WT♂ x WT⚥
Δ♂ x Δ⚥
C
WT♂ x WT⚥
Δ♂ x Δ⚥
D
WT♂ x WT⚥
Δ♂ x Δ⚥
E
x
DNA SPOT::SAS-4 SSNA-1::C-tag
Δ♂ x WT⚥
F
****
SPOT::SAS-1 signal intensity (arb. units)
0
100
200
300
WT ssna-1(Δ)
****
GFP::SAS-7 signal intensity (arb. units)
0
250
500
750
1000
WT ssna-1(Δ)
DNA SPOT::SAS-1 GFP::SAS-7
WT
ssna-1(Δ)

Fig. S5: Loss of *ssna-1* results in centriole fragmentation but not premature disengagement. **A-D** Representative images of embryos produced from *SPOT::sas-4* male x *sas-4::GFP* hermaphrodite matings. Insets are 2X magnifications. All images are maximum intensity projections. Scale bars = 10 µm. **A** WT x WT (top) and *ssna-1(Δ)* x *ssna-1(Δ)* embryos at mid anaphase. In WT x WT embryos, mother (magenta) and daughter (green) centrioles remain associated prior to normal disengagement in late anaphase/telophase while in *ssna-1(Δ)* x *ssna-1(Δ)* embryos, mother (magenta) centrioles fracture (centrosome 1). One of these two magenta centriole fragments remains associated with the green daughter centriole indicating the centriole pair is still engaged. **B** WT x WT (top) and *ssna-1(Δ)* x *ssna-1(Δ)* embryos during prophase and prometaphase. Note that all magenta centrioles and their green daughters are coincident and therefore engaged. Note also an instance of apparent centriole fragmentation of a green centriole in the *ssna-1(Δ)* embryo (centrosome 2). **C and D** Representative images of one- (C) and two-cell (D) embryos showing that in both WT x WT (top) and *ssna-1(Δ)* x *ssna-1(Δ)* embryos all magenta paternally derived centrioles remain engaged with their green daughters through early anaphase (WT = 64 centrosomes and *ssna-1(Δ)* = 56 centrosomes). **E** SSNA-1 from the maternal cytoplasm is recruited to sperm centrioles lacking SSNA-1 early in meiosis. Shown is an embryo produced by mating *SPOT::sas-4*; *ssna-1(Δ)* males to *ssna-1::C-tag* hermaphrodites. SSNA-1::C-tag (green) is detected at the sperm-derived centriole marked by SPOT::SAS-4 (magenta). A similar observation was made in all nine meiotic stage embryos scored. **F** The centriole levels of SAS-1 are reduced in the absence of SSNA-1. Wild-type and *ssna-1(Δ)* embryos strained for endogenously tagged SPOT::SAS-1 and GFP::SAS-7. Note that SPOT::SAS-1 also forms satellite-like structures in wild-type embryos. Also note that in the *ssna-1* mutant SPOT::SAS-1 localization to the satellite-like structures is eliminated while the levels of SPOT::SAS-1 at centrioles is markedly reduced. Quantitation of SPOT::SAS-1 and GFP::SAS-7 signal intensities is on the left. Each datapoint represents the intensity of single centriole. Mean and SD are shown. For SPOT::SAS-1, n= 105 (WT) and 118 (*ssna-1(Δ)*). For GFP::SAS-7, n= 105 (WT) and 118 (*ssna-1(Δ)*). ****$p<0.0001$ as determined by an unpaired two-tailed Student's *t* test.

Movie S1: SSNA-1 satellite-like structures display dynamic behavior. Time-lapse video corresponding to Fig. 2E of an embryo expressing SSNA-1::wrmScarlet, GFP::histone, and γ-tubulin::GFP. A side view of the ABpl blastomere dividing is shown. SSNA-1 satellites display dynamic behavior during division whereby they disperse at the end of mitosis following PCM breakdown and spread out across the nucleus. As the cell progresses through mitosis, the satellites accumulate around the centrosome of each pole. Maximum accumulation is achieved by late metaphase/early anaphase.

Source Data Files: Supplementary Figure 4A

sas-6			
smd-1 RNAi	*sas-6* RNAi	WT	*ssna-1(Δ)*
0.82836376	0.1429905	0.96588732	0.74794931
		1.27784944	0.77354017
		0.75626324	0.75930528

Lane	Sample
1	*smd-1* RNAi
2	sas-6 RNAi
3	WT
4	WT
5	WT
6	ssna-1(Δ)
7	ssna-1(Δ)
8	ssna-1(Δ)

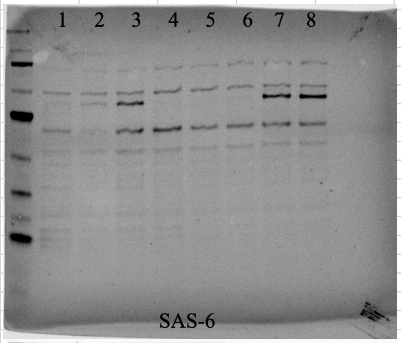

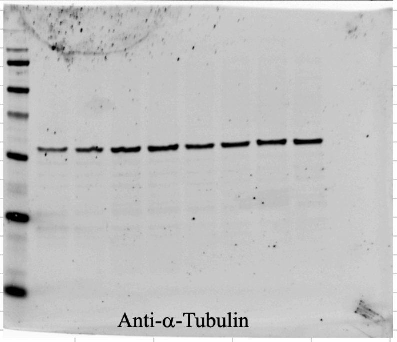

	sas-5					
	Isoform A			Isoform C		
smd-1 RNA	1.56776743			1.50737993		
sas-5 RNAi	0.52249772			0.44263294		
WT	0.89415963	0.97545561	1.13038476	1.00342327	0.98452525	1.01205147
ssna-1(Δ)	0.78793102	0.73118211	0.84735328	0.5629834	0.71994596	0.80185296

Lane	Sample
1	*smd-1* RNAi
2	sas-6 RNAi
3	WT
4	WT
5	WT
6	ssna-1(Δ)
7	ssna-1(Δ)
8	ssna-1(Δ)

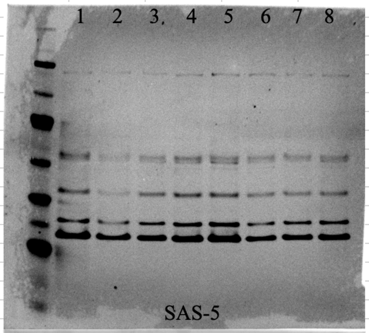

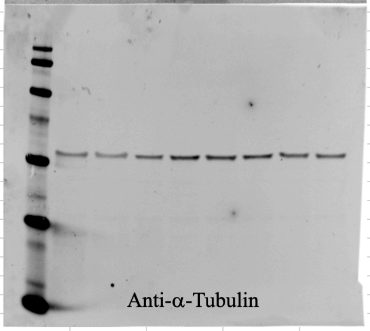

Supplement: Supplementary file 1 — Supplementary Information [file 41467_2025_59939_MOESM1_ESM.pdf]
