# Supplementary material for: C. elegans SSNA-1 is required for the structural integrity of centrioles and bipolar spindle assembly
Source: Nat Commun. 2025 Jun 5;16:5220. doi: 10.1038/s41467-025-59939-0 (PMC12141670; doi:10.1038/s41467-025-59939-0)
Supplement: Supplementary file 2 — Description of Additional Supplementary Files [file 41467_2025_59939_MOESM2_ESM.pdf]

Description of Additional Supplementary Files

File Name: Supplementary Movie 1
Description: **SSNA-1 satellite-like structures display dynamic behavior.** Time-lapse video corresponding to Fig. 2E of an embryo expressing SSNA-1::wrmScarlet, GFP::histone, and gtubulin::GFP. A side view of the ABpl blastomere dividing is shown. SSNA-1 satellites display dynamic behavior during division whereby they disperse at the end of mitosis following PCM breakdown and spread out across the nucleus. As the cell progresses through mitosis, the satellites accumulate around the centrosome of each pole. Maximum accumulation is achieved by late metaphase/early anaphase.
