# Supplementary material for: C. elegans SSNA-1 is required for the structural integrity of centrioles and bipolar spindle assembly
Source: Nat Commun. 2025 Jun 5;16:5220. doi: 10.1038/s41467-025-59939-0 (PMC12141670; doi:10.1038/s41467-025-59939-0)
Supplement: Supplementary file 4 — Reporting Summary [file 41467_2025_59939_MOESM4_ESM.pdf]

nature portfolio

Corresponding author(s): Kevin F O'Connell, Naoko Mizuno

Last updated by author(s): Apr 23, 2025

# Reporting Summary

Nature Portfolio wishes to improve the reproducibility of the work that we publish. This form provides structure for consistency and transparency in reporting. For further information on Nature Portfolio policies, see our Editorial Policies and the Editorial Policy Checklist.

## Statistics

For all statistical analyses, confirm that the following items are present in the figure legend, table legend, main text, or Methods section.

| n/a | Confirmed | |
|---|---|---|
| ☐ | ☒ | The exact sample size (*n*) for each experimental group/condition, given as a discrete number and unit of measurement |
| ☐ | ☒ | A statement on whether measurements were taken from distinct samples or whether the same sample was measured repeatedly |
| ☐ | ☒ | The statistical test(s) used AND whether they are one- or two-sided<br>*Only common tests should be described solely by name; describe more complex techniques in the Methods section.* |
| ☒ | ☐ | A description of all covariates tested |
| ☐ | ☒ | A description of any assumptions or corrections, such as tests of normality and adjustment for multiple comparisons |
| ☐ | ☒ | A full description of the statistical parameters including central tendency (e.g. means) or other basic estimates (e.g. regression coefficient) AND variation (e.g. standard deviation) or associated estimates of uncertainty (e.g. confidence intervals) |
| ☐ | ☒ | For null hypothesis testing, the test statistic (e.g. *F*, *t*, *r*) with confidence intervals, effect sizes, degrees of freedom and *P* value noted<br>*Give P values as exact values whenever suitable.* |
| ☒ | ☐ | For Bayesian analysis, information on the choice of priors and Markov chain Monte Carlo settings |
| ☒ | ☐ | For hierarchical and complex designs, identification of the appropriate level for tests and full reporting of outcomes |
| ☒ | ☐ | Estimates of effect sizes (e.g. Cohen's *d*, Pearson's *r*), indicating how they were calculated |

*Our web collection on statistics for biologists contains articles on many of the points above.*

## Software and code

Policy information about availability of computer code

| | |
|---|---|
| Data collection | Nikon NIS Elements AR 5.21.03 64-bit software was used to collect images of live and fixed specimens on the confocal microscope.<br>Images of gels were obtained with Invitrogen iBright 1500 software |
| Data analysis | Statistical analysis was performed using Prism 10 for MacOS version 10.2.2<br>Image J2 version 2.14.0/1.54f was used to make fluorescence intensiy measurements |

For manuscripts utilizing custom algorithms or software that are central to the research but not yet described in published literature, software must be made available to editors and reviewers. We strongly encourage code deposition in a community repository (e.g. GitHub). See the Nature Portfolio guidelines for submitting code & software for further information.

## Data

Policy information about availability of data

All manuscripts must include a data availability statement. This statement should provide the following information, where applicable:

- Accession codes, unique identifiers, or web links for publicly available datasets
- A description of any restrictions on data availability
- For clinical datasets or third party data, please ensure that the statement adheres to our policy

All source data are available in the figshare repository.

## Research involving human participants, their data, or biological material

Policy information about studies with human participants or human data. See also policy information about sex, gender (identity/presentation), and sexual orientation and race, ethnicity and racism.

| | |
|---|---|
| Reporting on sex and gender | *Use the terms sex (biological attribute) and gender (shaped by social and cultural circumstances) carefully in order to avoid confusing both terms. Indicate if findings apply to only one sex or gender; describe whether sex and gender were considered in study design; whether sex and/or gender was determined based on self-reporting or assigned and methods used.* *Provide in the source data disaggregated sex and gender data, where this information has been collected, and if consent has been obtained for sharing of individual-level data; provide overall numbers in this Reporting Summary. Please state if this information has not been collected.* *Report sex- and gender-based analyses where performed, justify reasons for lack of sex- and gender-based analysis.* |
| Reporting on race, ethnicity, or other socially relevant groupings | *Please specify the socially constructed or socially relevant categorization variable(s) used in your manuscript and explain why they were used. Please note that such variables should not be used as proxies for other socially constructed/relevant variables (for example, race or ethnicity should not be used as a proxy for socioeconomic status).* *Provide clear definitions of the relevant terms used, how they were provided (by the participants/respondents, the researchers, or third parties), and the method(s) used to classify people into the different categories (e.g. self-report, census or administrative data, social media data, etc.)* *Please provide details about how you controlled for confounding variables in your analyses.* |
| Population characteristics | *Describe the covariate-relevant population characteristics of the human research participants (e.g. age, genotypic information, past and current diagnosis and treatment categories). If you filled out the behavioural & social sciences study design questions and have nothing to add here, write "See above."* |
| Recruitment | *Describe how participants were recruited. Outline any potential self-selection bias or other biases that may be present and how these are likely to impact results.* |
| Ethics oversight | *Identify the organization(s) that approved the study protocol.* |

Note that full information on the approval of the study protocol must also be provided in the manuscript.

# Field-specific reporting

Please select the one below that is the best fit for your research. If you are not sure, read the appropriate sections before making your selection.

☒ Life sciences ☐ Behavioural & social sciences ☐ Ecological, evolutionary & environmental sciences

For a reference copy of the document with all sections, see nature.com/documents/nr-reporting-summary-flat.pdf

# Life sciences study design

All studies must disclose on these points even when the disclosure is negative.

| | |
|---|---|
| Sample size | For comparrson of live and dead embryos based on past experience we have chosen a rnimal size of 9 biological replicates. For westerns we analyse a minimum of three biological replicates which is standard in our field. |
| Data exclusions | no exclusions |
| Replication | All assays were performed a minimum of three times and in all cases the results were found to be reproducible |
| Randomization | Worms of a given genotype were randomly picked from different plates for each repetition of a given assay |
| Blinding | Blinding was not performed. In all of our assays the results were not open to subjective nterpretation. For instance, in embryo viability assays, a given embryo was either dead (unhatched egg) or alive. |

# Reporting for specific materials, systems and methods

We require information from authors about some types of materials, experimental systems and methods used in many studies. Here, indicate whether each material, system or method listed is relevant to your study. If you are not sure if a list item applies to your research, read the appropriate section before selecting a response.

## Materials & experimental systems

| n/a | Involved in the study |
| --- | --- |
| ☐ | ☒ Antibodies |
| ☒ | ☐ Eukaryotic cell lines |
| ☒ | ☐ Palaeontology and archaeology |
| ☐ | ☒ Animals and other organisms |
| ☒ | ☐ Clinical data |
| ☒ | ☐ Dual use research of concern |
| ☒ | ☐ Plants |

## Methods

| n/a | Involved in the study |
| --- | --- |
| ☒ | ☐ ChIP-seq |
| ☒ | ☐ Flow cytometry |
| ☒ | ☐ MRI-based neuroimaging |

# Antibodies

| | |
| --- | --- |
| Antibodies used | anti-SAS-4 rabbit polyclonal (custom production) 1:1000 dilution<br>anti-SSNA-1 rabbit polyclonal (custom production) 1:250-1:1000 dilution<br>anti-alpha-tubulin DMIA (Millipore Sigma) cat# CP06 1:1000 dilution<br>Alexa Fluor 488 anti-rabbit (ThermoFisher Scientific ) cat# A32731, 1:1000 dilution<br>Alex Fluor 568 anti-mouse (ThermoFisher Scientific) cat# A-11004, 1:1000 and 1:400 dilutions<br>Alex Fluor 488 anti-rabbit (ThermoFisher Scientific) cat# A-11008, 1:400 dilution<br>Alexa Fluor 488 anti-rat (ThermoFisher Scientific) (cat# A21208), 1:400 dilution<br>Alexa Fluor 568 anti-guinea pig (ThermoFisher Scientific) cat # A11075, 1:400 dilution<br>anti-guinea pig Cy5 (Jackson ImmunoResearch) cat # 706-175-148, 1:400 dilution<br>SPOT-Label Alexa Fluor 568 (Proteintech) cat# ebAF568, 1:1000 dilution<br>CaptureSelect Alexa Fluor anti-C-tag antibody (ThermoFisher Scientific ) cat# 7213252100, 1:1000 dilution<br>anti-alpha tubulin antibody (ABCD Antibodies) cat# ABCD_AA345, 1:250 dilution<br>anti-beta tubulin antibody (ABCD Antibodies) cat# ABCD_AA344, 1:250 dilution<br>anti-HA antibody (Millipore Sigma) cat# 11867423001 , 1:250 dilution<br>mouse monoclonal anti-FLAG (Millipore Sigma), cat# F1804, 1:250 dilution<br>rabbit anti-GFP antibody (Torry Pines Biolabs) cat # TP401, 1:250 dilution<br>rabbit polyclonal anti-SAS-5 (custom production), 1:1000 dilution<br>guinea pig polyclonal anti-SAS-6 (custom production), 1:1000 dilution<br>anti-mouse 680 IRDye Seconday (LiCOR Biosciences) cat# 926-68070, 1:14,000 dilution<br>anti-rabbit 800 IRDye Seconday (LiCOR Biosciences) cat# 926-32211 1:14,000 dilution<br>anti-guinea pig 800 IRDye Seconday (LiCOR Biosciences) cat# 926-32411, 1:14,000 dilution |
| Validation | Where appropriate, each antibody was validated by staining specimens that lacked the epitope against which the antibody was raised. In all cases the antibody only produced a signal in strains expressing the target epitope |

# Animals and other research organisms

Policy information about studies involving animals; ARRIVE guidelines recommended for reporting animal research, and Sex and Gender in Research

| | |
| --- | --- |
| Laboratory animals | C. elegans |
| Wild animals | none |
| Reporting on sex | Sex was irrelevant as nearly all of the data was was based on analysis of early embryos. Some analysis of the female germline was performed and this is explicitly stated in the paper. |
| Field-collected samples | n/a |
| Ethics oversight | n/a |

Note that full information on the approval of the study protocol must also be provided in the manuscript.

## Plants

| | |
|---|---|
| Seed stocks | *Report on the source of all seed stocks or other plant material used. If applicable, state the seed stock centre and catalogue number. If plant specimens were collected from the field, describe the collection location, date and sampling procedures.* |
| Novel plant genotypes | *Describe the methods by which all novel plant genotypes were produced. This includes those generated by transgenic approaches, gene editing, chemical/radiation-based mutagenesis and hybridization. For transgenic lines, describe the transformation method, the number of independent lines analyzed and the generation upon which experiments were performed. For gene-edited lines, describe the editor used, the endogenous sequence targeted for editing, the targeting guide RNA sequence (if applicable) and how the editor was applied.* |
| Authentication | *Describe any authentication procedures for each seed stock used or novel genotype generated. Describe any experiments used to assess the effect of a mutation and, where applicable, how potential secondary effects (e.g. second site T-DNA insertions, mosiacism, off-target gene editing) were examined.* |
